# Headache - A Window to Dementia: An Unexpected Twist

**DOI:** 10.7759/cureus.13398

**Published:** 2021-02-17

**Authors:** Shayka Sharif, Amber Saleem, Evgenia Koumadoraki, Sommer Jarvis, Nikolaos Madouros, Safeera Khan

**Affiliations:** 1 Internal Medicine, California Institute of Behavioral Neurosciences & Psychology, Fairfield, USA; 2 Family Medicine, California Institute of Behavioral Neurosciences & Psychology, Fairfield, USA; 3 Pathology, California Institute of Behavioral Neurosciences & Psychology, Fairfield, USA; 4 Anatomy/Cell Biology, California Institute of Behavioral Neurosciences & Psychology, Fairfield, USA; 5 Surgery, California Institute of Behavioral Neurosciences & Psychology, Fairfield, USA

**Keywords:** headache and dementia, migraine and dementia, primary headache and dementia, headache and cognitive decline, primary headache and cognitive decline, migraine and cognitive decline, migraine and antidepressants

## Abstract

Migraine and other types of headaches have several symptoms associated with them. The association between migraine and dementia has been considered. It is a topic of discussion and appears to be multifactorial. Dementia is a cluster of symptoms, with memory loss and cognitive dysfunction being the prominent symptoms. In this review, we discussed the association of headache and cognitive dysfunction in a broader context and how the practiced treatment of headaches may silently lead to dementia. We conducted a thorough literature search using PubMed as our main database. The articles exploring the association between headache (both migraine and non-migraine) and dementia were included. Some risk factors like migraine-induced stroke and inherent vascular diseases in migraine patients channeling to stroke and dementia were not considered. A total of 28 studies were included for review. All the reviewed studies put together showed an association between headache and cognitive dysfunction of any form. They showed that the frequency and duration of headache is a determinant for dementia. Few studies also focused on how treating headaches with certain drugs can lead to dementia. The reviewed published literature showed that headaches of any sort and their treatment are potentially linked to dementia. Not all headache patients will require medical treatment, as the benefit might outweigh the risk sometimes. It is interim to understand these facts and formulate a better protocol for treating headache patients. However, due to some discordant results, further studies are needed.

## Introduction and background

"Of those at least 65 years of age, there is an estimated five million adults with dementia in 2014 and projected to be nearly 14 million by 2060" [1].

Dementia is a neurological disease that includes a cluster of symptoms involving memory, thought process, language, and problem-solving and task-performing capacities. It is debilitating enough to interfere with daily life activities. Dementia occurs due to a number of causes. There are various types of dementia. Alzheimer's disease (AD) and vascular dementia (VD) are the two leading causes of dementia [2]. On the other hand, primary headache disorders are several diseases with different characteristics. The most common ones are migraines with or without aura, tension-type headache (TTH), and cluster headache. Worldwide, roughly 45% of adults in the general population are victims of headache disorders [3]. The link between symptoms such as headache and the clinical presentation of dementia is seldom a coincidence [4]. The registry of the years back and population-based cohort studies have successfully shown the potential association that migraine and dementia might have [5].

Although only a scarce number of studies had been conducted about the link between non-migrainous headache and dementia, with only two population-based studies in Norway, there was a potential association between the two entities [6]. Moreover, some studies from previous years reported that 80% of individuals with a non-migrainous headache might be assumed to have a TTH [7]. Despite being the most common type, TTH and other non-migrainous headaches have not come into the research limelight.

Non-migrainous headaches and vascular diseases are also interconnected [8]. These vascular risk factors are also prevalent in migraine headaches and may potentiate the risk of dementia. Therefore, primary headaches and headaches disorders can be arguably believed to lead to an increased risk of dementia. Currently, no effective drugs have been identified that may significantly slow down the course of dementia [9]. Researchers have shifted their focus in studying the risk factors for dementia and expect to reduce the incidence of dementia by effectively controlling the potential risk factors.

There has been a scarce amount of studies focusing on the relationship between primary headache and a large and cognitive decline. It has mostly been migraine only that had been studied immensely. But other primary headache subtypes leading to dementia need to be studied as well. Our main focus in this study has been to compare and contrast the link between primary headache as a whole (both migraine and non-migraine) and all-cause-dementia in all age groups. One other sector that has been seldom studied is the risk of treating headache patients with antidepressants and nonsteroidal anti-inflammatory drugs (NSAIDs) that may aggravate dementia risks. Our study tries to shed some light on this unexplored aspect of the study as well.

## Review

Methods

Data were collected by using the PubMed search engine as the primary source. A number of articles were also gathered from Medline and ScienceDirect. The search was conducted using keywords "Migraine and Dementia," "Headache and Dementia," "Primary Headache and Dementia," "Headache and Cognitive Decline," "Migraine and Cognitive Decline," "Primary Headache and Cognitive Decline," "Antidepressants and Dementia," "Antidepressants and Cognitive Decline." The number of articles yielded with each keyword is summed up in Table 1 and Table 2. No guidelines were followed in particular.

**Table 1:** Number of articles yielded by keywords

Keywords	Database	Articles yielded
Migraine and Dementia	PubMed	131
Headache and Dementia	PubMed	151
Primary Headache and Dementia	PubMed	37
Migraine and Cognitive Decline	PubMed	86
Headache and Cognitive Decline	PubMed	194
Primary Headache and Cognitive decline	PubMed	26

**Table 2:** Number of articles yielded by keywords

Keywords	Database	Articles yielded
Antidepressants and Dementia	PubMed	537
Antidepressants and Cognitive Decline	PubMed	310

Studies and articles involving the link between headache and dementia were mostly included in the data. Those talking about migraine-induced stroke and genetic vascular diseases (such as CADASIL (cerebral autosomal dominant arteriopathy with subcortical infarcts and leukoencephalopathy)) sequentially leading to stroke in migraine patients were not included. Articles discussing antidepressants used in headache treatment or prophylaxis leading to dementia were used and not the general link between antidepressants and dementia. A few selective systematic reviews and meta-analyses were used. Other selection criteria used were: full-text articles, articles from the last five years, articles with human subjects only, and articles written in English.

The entire procedure was conducted ethically. No statistical analyses were conducted. Table 3 shows the inclusion and exclusion criteria.

**Table 3:** Inclusion and exclusion criteria

Inclusion criteria	Exclusion criteria
1) Articles from the last five years, 2) Full-text articles, 3) Abstract only articles, 4) Articles with human subjects, 5) Articles in English	1) Articles with non-human subjects, 2) Articles in any language other than English, 3) Articles with causes of dementia other than headache, 4) Migraine-induced cardiovascular accidents leading to dementia, 5) Genetic vascular diseases sequentially causing stroke in migraine patients

Results

We initiated our search with several keywords. "Migraine" generated eight 8249 results; "Dementia" resulted in 56516 articles. Simultaneously, other synonyms such as "Headache" assembled 21589 published research articles and "Cognitive Decline" generated 35121 results. Keywords "Migraine and Dementia," "Headache and Dementia," "Primary Headache and Dementia" generated the following number of results, respectively: 131, 151, 37. The rest of the keywords showed the following results: "Migraine and Cognitive Decline," "Headache and Cognitive Decline," "Primary Headache and Cognitive Decline," "Antidepressant and Dementia," "Antidepressant and Cognitive Decline": 86, 194, 26, 537, 310, respectively.

The articles were first sorted to eliminate any duplication, which was done by looking into the titles. Filters were applied to narrow down the search, including full-text articles, abstract only, human subjects, articles published in the last five years, and articles written in English. These were further sorted to keep the most relevant ones again by reading the titles. After all these procedures, 59 related articles were left. After reading 59 articles, the 32 most relevant articles were finalized for the review article. Two articles were not available, and two did not relate to the review desires.

**Figure 1:**
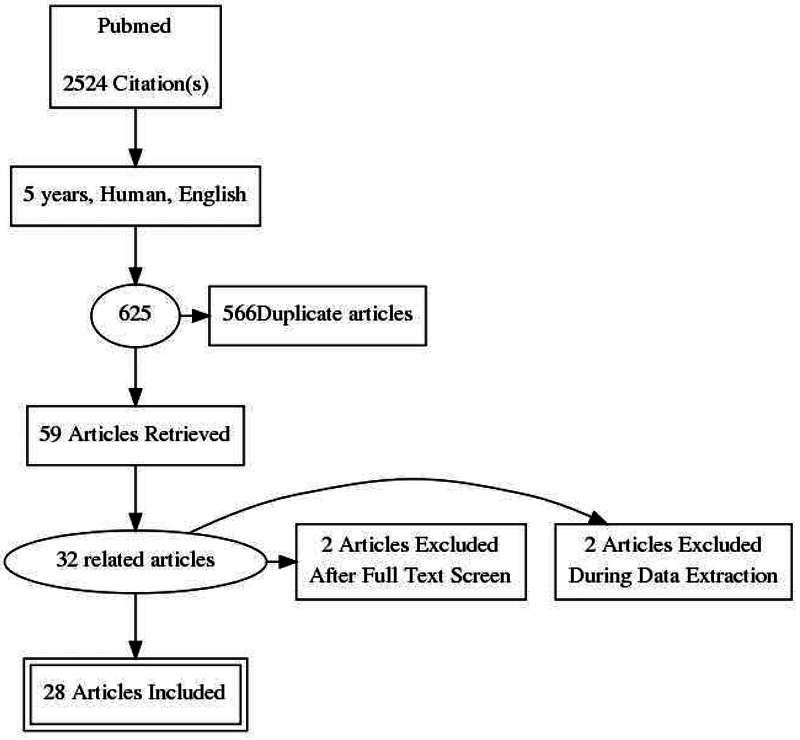
Data selection process

Out of those, we included 21 observational studies, four reviews, two meta-analyses, and one case report. The study selection process is shown in Figure 1.

The main characteristics of the included studies are summarized in Table 4.

**Table 4:** Main characteristics of the included studies

Article name	Author and year	Study type	Follow-up years	Headache type	Dementia type	Confounders adjusted
Migraine and the risk of all-cause dementia, Alzheimer's disease, and vascular dementia: A prospective cohort study in community-dwelling older adults	Rebecca E. Morton et al, 2019 [10]	Observational	Five	Migraine	All-cause Dementia	Age, gender, education, and depression
Headache disorder and the risk of dementia: a systematic review and meta-analysis of cohort studies	Jing Wang et al, 2018 [11]	Systematic review and Meta-analysis of Cohort studies	None	Headache disorder	All-cause dementia and AD	None
Mid- to late-life migraine diagnoses and risk of dementia: a national register-based follow-up study	Sabrina Islamoska et al, 2020 [12]	Cohort	Six years and nine months	Migraine	All-cause-dementia	Sex and birthdate
Age-specific and gender-dependent impact of primary headache disorders on dementia risk: Population-based longitudinal study	Jiu-Haw Yin et al, 2018 [13]	Observational	Five	Primary headache disorder	All-cause dementia	Age, sex, hypertension, diabetes mellitus, hyperlipidemia, IHDs, AF, TUD, alcoholism, obesity, PD, CVA, major depression, CKD, and CAI
Association between migraine headaches and dementia in more than 7,400 patients followed in general practices in the United Kingdom	Karel Kostev et al, 2019 [14]	Observational	Ten	Migraine	All-cause-dementia	Age, sex, index year, and co-diagnoses
Primary headaches in an elderly population seeking medical care for cognitive decline	Michele Feleppa et al, 2016 [15]	Observational	Ten	Headache disorders	Cognitive decline	None
Subjective cognitive complaints and migraine characteristics	Hsuan‐Te Chu et al, 2019 [16]	Observational	Nil	Migraine	Cognitive complaints	Not mentioned
Cognitive impairment in chronic migraine: a cross-sectional study in a clinic-based sample	Nina Latysheva et al, 2020 [17]	Observational	None	Chronic migraine	Cognitive decline	Not mentioned
Subjective cognitive symptoms during a migraine attack: a prospective study of a clinic-based sample	Isabel Pavão Martins et al, 2016 [18]	Observational	None	Migraine	attention difficulties diminished cognitive efficiency and Processing speed impairment.	Not mentioned
Duration and frequency of migraines affect cognitive function: evidence from neuropsychological tests and event-related potentials	Lifang Huang et al, 2017 [19]	Observational	None	Duration and frequency of migraine attack	Cognitive decline	Age
Cognition and cognitive impairment in migraine	Raquel Gil-Gouveia et al, 2019 [20]	Review	None	Migraine	Nothing conclusive	None
Neuropsychological assessment in migraine patients: a descriptive review of cognitive implications	Maria Foti et al, 2018 [21]	Review	None	Migraine	Discordant result	None
Chronic migraine patients show cognitive impairment in an extended neuropsychological assessment	Karen S Ferreira et al, 2018 [22]	Observational	None	Chronic migraine	Cognitive decline	Frequency and intensity of the headache, medication used, and associated comorbidities
Prospective memory is dysfunctional in migraine without aura	Gabriella Santangelo et al, 2018 [23]	Observational	None	Migraine without aura	Prospective memory dysfunction	None
Cognitive dysfunctions and psychological symptoms in migraine without aura	Gabriella Santangelo et al, 2016 [24]	Observational	None	Drug-naïve migraine without aura (MwoA)	Subtle cognitive dysfunctions	Age and education
Subjective cognitive decline in patients with migraine and its relationship with depression, anxiety, and sleep quality	Sun Hwa Lee et al, 2017 [25]	Observational	None	Migraine	Subjective cognitive decline	Gender, age, and education level
Functional connectivity and cognitive impairment in migraine with and without aura	Viviana Lo Buono et al, 2017 [26]	Observational	None	Migraine	Functional connectivity	Age and sex
Cognitive dysfunction and migraine	Doga Vuralli et al, 2018 [27]	Systemic review	None	Migraine attack	Attack related to poor cognition	None
Cognitive functioning in adolescents with migraine	Melissa Andréia Costa-Silva et al, 2016 [28]	Observational	None	Migraine	Cognitive impairment	Socioeconomic status
Cognitive Impairment in Children and Adolescents with Migraine	Cristiano Termine et al, 2018 [29]	Review	None	Migraine	Cognitive deficits	None
Headaches and risk of dementia	Nian-Sheng Tzeng et al, 2017 [30]	Observational	Ten	Migraine and tension-type headache	Non-vascular dementia	Age and sex, monthly income, urbanization level, geographic region of residence, and comorbidities
Increased risk of dementia in patients with tension-type headache: a nationwide retrospective population-based cohort study	Fu-Chi Yang et al, 2016 [31]	Observational	Ten	TTH	All-cause-dementia	Sex- and age
Cognitive performance and the alteration of neuroendocrine hormones in chronic tension‐type headache	Ping Qu et al, 2017 [32]	Observational	None	Chronic tension-type headache	Memory impairment	Age, sex, years of education, and medical history
Cognitive aging in migraine sufferers is associated with more subjective complaints but similar age-related decline: a 5-year longitudinal study	Isabel Pavão Martins et al, 2020 [33]	Observational	Five	Migraine and non-migrainous headache	None (more cognitive complaints)	Age, gender, literacy, and depressive symptoms
Recurrent reversible cognitive impairment in a cluster headache patient: a case report	Valentina Favoni et al, 2018 [34]	Observational	None	Cluster headache	Recurrent cognitive impairment	None
Increased risk of dementia in patients with antidepressants: a meta-analysis of observational studies	Yao-Chin Wang et al, 2018 [35]	Meta-analysis	None	Antidepressant use	Dementia: monoamine oxidase inhibitor therapy had a higher risk of developing dementia than those with tricyclic and selective serotonin reuptake inhibitor therapy	None
Antidepressants and risk of dementia in migraine patients: a population-based case-control study	Cynthia Wei-Sheng Lee et al, 2017 [36]	Observational	Six	Antidepressants	Dementia	Age, sex, year of migraine diagnosis, the year of the index date, diabetes, hypertension, stroke, coronary artery disease, head, injury, anxiety, depression
Cognitive decline in chronic migraine with nonsteroid anti-inflammation drug overuse	Xiaoying Cai et al, 2019 [37]	Observational	Four	Chronic migraine with medication overuse headache (CM-MOH), chronic migraine without medication overuse headache (CMwoMOH), and migraine without aura	Cognitive Decline	Comorbidity, headache, neurological examination

Discussion

The debilitating effects of migraines are very well-established. Multiple pathophysiologies can explain migraine and dementia. Migraine and stroke have been a topic of discussion very recently, and there is enough literature about it. Our primary focus was on the relationship between headache as a whole and dementia. We opted to highlight the consequences that primary headache has on cognition, leaving out mechanisms such as stroke and inherent vascular disease in migraine patients that lead to cognitive impairment. Our study indicates multiple headache-related risk factors that lead to cognitive dysfunction. While migraine is the most commonly discussed topic here, we also tried to talk about other headache subtypes and their treatment, resulting in cognitive decline. Below are some of the common factors discussed.

Migraine Headache

Among the primary headache types, migraine is the subtype that is studied much more than any others. Migraine is the most common type of neurological disorder in all age types. In a prospective cohort study of older adults in the community, AD and all‐cause dementia were observed in migrainers [10]. The evidence did not support an association between migraines and VD. Studies revealed that any history of headache in the general population may be a predictor of all-cause dementia. [11]. A Danish national register-based follow-up study conducted between 1935 and 1956 hypothesized that patients reporting migraine in midlife are likely to experience cognitive decline later in their life [12]. In a population-based longitudinal study, an age and gender-specific pattern has been found. It was seen that women and elderly patients (more than 65 years of age) were more prone to develop dementia [13]. Some studies revealed that elderly migrainers have a high possibility of developing AD and all-cause dementia [14-15]. A cross-sectional study was conducted on migraine attack frequency and the severity of dementia. It was found that the severity of memory decline is related to migraine frequency and duration [16]. A similar hypothesis was seen in several other articles [17-19]. Some studies found a discordant result of the migraine and dementia link [20-21]. A study focusing only on chronic migraine supported the idea that it causes neuroplasticity, resulting in maladaptation and cognitive dysfunction [22]. An observational study narrowed down the result of migraine without aura having only prospective memory dysfunction [23]. Migraine without aura patients have a range of cognitive complaints. Some of these complaints are anxiety, depression, and poor sleep quality [24-25]. Another study pinpointed a neurological level of migraine on the brain, i.e., the insular cortex develops altered connectivity. The insula is related to cognition, which proves the concern of the link between migraine and dementia as well [26]. The migraine attack period shows more cognitive impairment than migraine-free periods [27]. The results of research studies to date show that children and adolescents affected by migraines may present specific cognitive deficits [28]. Children suffering from migraines, aged six to 12 years, and their unaffected siblings showed no significant difference in cognition [29].

*Other Non-Migraine Headaches* 

Non-migraine headaches have been seldom studied, and a few studies show discordant results in them. One of the studies identifies how migraine and non-migraine headaches can result in VD. From the same study, it could also be inferred that non-migraine headaches are linked to both VD and mixed dementia. When comparing primary headache disorders (PHDs) with non-PHDs, it was identified that sufferers of PHD did not have an increased VD, a result in contradiction to previous studies. White matter (WM) hyper-densities had also been observed in non-migrainous headaches. These WM hyper-densities, like any other brain infarcts (ischaemic stroke), increase the likelihood of dementia [13].

a) Tension-type headache (TTH): It is yet another non-migraine headache subtype that increases the risk of non-vascular dementia but is not a predictor for vascular dementia, one of the studies concluded [30]. In a cross-sectional study of headache prevalence in elderly patients with dementia, TTH was the commonest found type. There was a convincing significance of this type leading to dementia. One very recent longitudinal study found out that TTH patients had no apparent risk of AD and VD, which reflects the results in previous studies that headache disorder does not lead to AD in later life. Similarly, many people who developed dementia later in life reported a history of headaches in their lifetime. Some of these results are opposite to a lot of studies conducted earlier. The reason for these differences is unknown. Pain pathways and memory networks are closely related and may explain the interconnection between chronic headaches and cognitive dysfunction [31]. A cross-sectional study conducted with the same motive backed up this theory. It also established neuroendocrine hormones to be linked with the chronic tension-type headache (CTTH) symptoms. Thus, it may be suggested that neuroendocrine hormones may play a vital role in the treatment of CTTH [32].

In another study, it has been seen that non-migraine headaches may influence some measures of executive performance. These headaches are not associated with an increased risk of cognitive decline, suggesting that repeated migraine attacks do not have a long-term impact on cognition. However, patients with migraines tend to report more subjective cognitive complaints during aging [33].

b) Cluster headache: It is another headache subtype that might also be a contributing factor for dementia. A case study reported a recurrent, reversible cognitive decline in a patient with cluster headache. However, the decline didn't persist beyond the episodes [34]. Further studies are required in a large population to hypothesize that cluster headache can be considered another cause of dementia.

Side-Effects of Drugs Used to Treat Headache

Antidepressants such as tricyclic antidepressants (TCA) and NSAIDs are some established means to treat headaches. Most recently, questions have been raised as to whether these treatments are silent causes of dementia. Few studies have been conducted on this topic, but it is a significant concern, mostly for elderly patients. The pathophysiology of antidepressants leading to dementia is seldom known. It is necessary to outweigh the risks and benefits of this mode of headache treatment.

a) Antidepressants in patients with depression: In a meta-analysis conducted on antidepressants and dementia, it was suggested that there were significant risks of dementia with antidepressant therapy [35]. Conclusions could be drawn as such TCAs benefit the patients by reducing the risk of dementia, whereas selective serotonin reuptake inhibitors (SSRIs) and newer non-SSRI antidepressants potentiate the risk of dementia in depression patients [36].

b) Antidepressant and migraine patients: TCA is the only known antidepressant used in migraine so far. There is very little information available on other subtypes of antidepressants such as SSRI and new generation antidepressants (NGA). A population-based case-control study has been reviewed to come to some inferences. Antiplatelet and proﬁbrinolytic properties are prevalent in SSRIs. Thus, SSRI is more likely to prevent migraine-induced vascular events such as subclinical infarction. It could be assumed in some studies that NGA medications potentiate dementia in migraine as well as depression patients. Other studies have found a contradictory result. Amitriptyline, a TCA, is very commonly used for migraine. It has anticholinergic properties and is likely to cause cognitive decline. Studies conducted showed SSRIs and TCAs having opposite results to those conducted earlier in patients with depression (decreased risk with SSRIs and no effect with TCAs) [36]. For better understanding, the action of antidepressants is shown in Table 5.

**Table 5:** Effects of different drugs on cognitive function SSRI: selective serotonin reuptake inhibitor; TCA: tricyclic antidepressant; NGA: new generation antidepressant

Drug	Antidepressants in Migraine	Antidepressants in Depression
SSRIs	Decreases Dementia	Increases Dementia
TCA	No Effect	Decreases Dementia
NGA	Increases Dementia	Increases Dementia

c) NSAID use in migraine: The chronic pain in migraine patients may damage cognitive function by two mechanisms. One could be the effect of prostaglandin itself. Second, an elevated level of prostaglandin downgrades the washout of amyloid-β. NSAID does its part by inhibiting the generation of prostaglandin and cutting off this lethal cascade. Although NSAIDs' effect on cognition is still unclear, some studies found its positive role in cognition [37].

Limitations

Even with all attempts put into this paper, we could not cut down some of our limitations. We did not have full-text access to some of the articles, so we could not make the best use of them. Some studies showed heterogeneous and contradictory results. These need more focused and mass studying to come to something conclusive. Finally, some shared risk factors, such as migraine-induced stroke and inherent vascular diseases in migraine patients, which leads to stroke and dementia, have not been included in our studies.

## Conclusions

This review was conducted with the motive of understanding the influencing factors of headache leading to dementia. We have critically discussed the various subtypes of primary headache and the treatment side effects of antidepressants and NSAIDs. This review deduced that both migraines with and without aura are contributing factors to cognitive decline. Migraine attack frequencies are also a determinant of dementia, which has been discussed as well. Overall, there were some discordant results, which means further studies need to be conducted for better understanding. Non-migraine headaches have been studied very little, to date. We came across very few data and tried making the best use of those to explain how non-migraine headaches may also lead to dementia. The reviewed studies reflected that non-migraine headaches, such as TTH and cluster headache, contribute to cognitive decline.

Some drug treatments used for headaches are assumed to lead to dementia. Antidepressants such as SSRI decreases dementia risk, TCA does not affect while NGA increases dementia risk. This has been discussed as much as possible with the limited data available. Our review aimed to put together all the information to understand how exactly migraine and dementia work. It has given insight into less-studied areas, such as dementia treatment, as the silent cause. This study will help physicians generate universal treatment protocols for headaches and avoid those that might increase the chances of dementia. It will help us understand how controlling the frequency of migraine attacks will prevent dementia.
